# Relationships between cerebral blood flow and cognition in young adults born very preterm and at term

**DOI:** 10.1038/s41598-026-49390-6

**Published:** 2026-04-21

**Authors:** Chandelle L. Piazza, Vanessa Schmithorst, Claire E. Kelly, Terrie E. Inder, Lex W. Doyle, Deanne K. Thompson, Leona Pascoe, Michael Takagi, Peter J. Anderson

**Affiliations:** 1 School of Psychological Sciences, Turner Institute for Brain and Mental Health, Monash University, Victoria, Australia; 2 Clinical Sciences, Murdoch Children’s Research Institute, Victoria, Australia; 3 Department of Radiology, University of Pittsburgh, Pittsburgh, PA USA; 4 Developmental Imaging, Murdoch Children’s Research Institute, Victoria, Australia; 5 Center for Perinatal and Infant Research, Rady Children’s Health, Orange County, Orange, CA USA; 6 Department of Pediatrics, School of Medicine, University of California Irvine, Irvine, CA 92697 USA; 7 Newborn Research, Royal Women’s Hospital, Victoria, Australia; 8 Department of Obstetrics, Gynaecology and Newborn Health, University of Melbourne, Victoria, Australia; 9 Department of Paediatrics, University of Melbourne, Victoria, Australia; 10 The Centre for Community Child Health, Policy and Equity, Murdoch Children’s Research Institute, Victoria, Australia; 11 Melbourne School of Psychological Sciences, University of Melbourne, Victoria, Australia; 12 Brain and Mind Research Group, Murdoch Children’s Research Institute, Victoria, Australia; 13 CHOC Research Institute, 1201 W. La Veta, Orange, CA 92868 USA

**Keywords:** Cerebral blood flow, Very preterm, Cognition, ASL-MRI

## Abstract

The mechanisms underpinning persistent cognitive challenges in young adults born very preterm (VP; < 32 weeks’ gestation) are unclear, but may include altered cerebral blood flow (CBF). We explored whether CBF differed between adults born VP and at term, and the associations between CBF and cognitive outcomes. Seventy-two participants born VP and 16 term-born controls born in Victoria, Australia, completed Arterial Spin Labelling-MRI and a neuropsychological assessment at 20 years of age. Using a voxel-wise general linear model, we analyzed CBF differences between VP and term-born individuals, and the relationships between CBF and cognitive outcomes. Adults born VP displayed lower CBF in several subcortical regions compared with their term-born peers. For both birth groups combined, CBF was positively associated with verbal delayed memory in frontal and temporal cortical regions, and negatively associated with executive functioning in frontal, temporal, occipital cortical regions and the cerebellum. Compared with the VP group, term-born adults exhibited stronger negative relationships between CBF and IQ, receptive language, and aspects of executive functioning. Few CBF-cognition relationships were observed in the VP group and when present, were positive. Overall, adults born VP exhibited lower CBF in subcortical regions with cortical CBF-cognition relationships in opposite directions across VP and term groups. These disparities may reflect cerebral dysmaturation in the VP group.

**Supplementary Information:**

The online version contains supplementary material available at 10.1038/s41598-026-49390-6.

## Introduction

Individuals born very preterm (VP; < 32 weeks’ gestation) face increased risks of poorer cognitive outcomes compared with term-born peers^1–4^, with these difficulties persisting into early adulthood^5,6^. However, the mechanisms underpinning these challenges are not well understood^7,8^.

A potential mechanism contributing to poorer cognitive functioning in those born VP is altered cerebral blood flow (CBF). Resting-state CBF can be measured using Arterial Spin Labelling (ASL), which uses radiofrequency pulses to magnetically label water in arterial blood to quantify CBF^9,10^.

Associations between resting-state CBF and cognition have been reported. Evidence from healthy adult, clinical (e.g., Alzheimer’s), and ageing populations have demonstrated that lower resting-state CBF is associated with poorer cognitive outcome across global^11–13^ (e.g., IQ) and specific cognitive domains, such as verbal memory^11–14^ and executive functioning^12,13,15,16^. Altered CBF has also been reported to be positively and negatively associated with cognition in healthy^17^ and clinical^18–20^ pediatric populations. For example, in children and adolescents with congenital heart disease, reduced resting-state CBF in medial frontal-parietal regions mediated poorer crystallised intelligence, while reduced CBF in lateral fronto-subcortical regions mediated better fluid intelligence compared with controls^20^. Similar findings have been observed in typically developing children (7–17 years), with the direction of relationships between CBF and IQ varying across brain regions^17^.

Altered CBF (either higher or lower depending on the region) has been reported in infants born preterm relative to term-born infants^21,22^, and correlated with later cognitive development at 18-months^23^. In young adults born very low birthweight (VLBW; < 1500 g) and a mean gestational age of 28.8 weeks, lower regional grey matter CBF in temporal and subcortical regions relative to term-born controls has been reported^24^, while Hijman et al. (2024) found positive relationships between voxel-wise subcortical, brainstem and white matter CBF and executive functioning in a combined cohort of children and adolescents born VP and at term^25^. Despite some initial evidence of altered CBF in young adults and brain-behavior relationships in adolescents born VP/VLBW, there is yet to be an investigation of CBF—cognition relationships in young adults born VP, and across multiple cognitive domains that support daily functioning.

This study aimed to 1) describe differences in CBF between 20-year-olds born VP and at term, and 2) describe the relationships between CBF and cognitive outcomes in these birth groups. Based on previous research^11,12,15,17,19,25,26^, we hypothesized that 1) individuals born VP would have lower CBF than term-born controls at 20-year of age, particularly in sub-cortical and temporal regions, and 2) lower CBF in frontal, temporal, parietal and subcortical regions would be associated with poorer cognitive performance in the domains of general intellect (IQ), processing speed (time it takes for an individual to interpret and respond to information)^27^, receptive language (understanding and comprehending verbal output), verbal learning and memory (encoding, storing and retrieving verbal information)^28^, and executive functioning (higher-order skills for goal-directed behaviour, such as planning, working memory, and cognitive flexibility)^29^ in both VP and term-born adults.

## Materials and methods

### Participants

Participants were from the Victorian Infant Brain Study (VIBeS) prospective longitudinal cohort of 224 children born VP (< 30 weeks’ gestation or with birthweight < 1250 g) recruited from the Royal Women’s Hospital, Melbourne, Victoria, between 2001 and 2003. 77 term-born controls (37 to 41 completed weeks’ gestation) were recruited, of whom 46 were recruited at birth from the Royal Women’s Hospital, Melbourne, Victoria, and 31 were recruited at 2 years of age from Maternal and Child Health Centres across Victoria. Individuals identified with congenital anomalies associated with adverse neurological outcome were excluded. One term-born control was later excluded due to the diagnosis of a congenital disorder. Further details regarding the VIBeS cohort can be found in previous publications of this cohort^30,31^.

At the 20-year follow-up of the VIBeS cohort, 136 VP and 46 term-born controls consented to participate in cognitive, cardiovascular and MRI measures. Social risk was dichotomised into higher and lower social risk groups based on six factors, including family structure, maternal age at birth, language spoken at home, education of primary caregiver, occupation of primary income earner, and employment status of primary income earner^32,33^. The study was approved by the Human Research Ethics Committee (HREC) at the Royal Children’s Hospital, Melbourne, with all procedures conducted in accordance with the HREC approval. Informed consent was obtained from participants via the Research Electronic Data Capture (REDCap).

### Neuropsychological assessment

Cognitive assessments were administered by trained examiners who were unaware of the birth group of the participants. A description of the domains, measures and outcome variables are provided in Supplementary Table 1. Briefly, IQ was estimated using the three-subtest Kaufman Brief Intelligence Test, second edition (KBIT-2)^34^, receptive language estimated using the Verbal Knowledge subtest from the KBIT-2, processing speed measured using Cogstate: Identification Test (CogState Ltd, Melbourne, Australia), verbal learning and memory using the California Verbal Learning Test, third edition (CVLT-3)^35^, and elements of executive functioning were assessed using The Tower Test (planning)^36^, Digit Span Backwards (working memory)^37^, and Contingency Naming Test (cognitive flexibility)^38^. Each cognitive variable was standardized to a z-score, relative to the mean and SD of the combined cohort (VP and term-born controls).

### Imaging procedure

Brain MRI scans were acquired at the Royal Children’s Hospital using a 3-Tesla Siemens MAGNETOM Prisma scanner with a 32-channel head coil. ASL sequences were acquired as part of a larger 1-h MRI protocol that included T1-weighted, T2-weighted, diffusion weighted imaging (DWI) and resting state functional MRI (rs-fMRI) sequences. During the ASL acquisition, participants were instructed to focus on a cross presented on a blank screen. The sequence used was based on the Human Connectome Project (HCP) protocol for 8–21-year-old participants^39^. The Pseudo-continuous arterial spin labelling (PC-ASL) images were acquired with parameters as follows: 2D EPI; voxel size 3 mm isotropic; axial slices; label duration: 1500 ms; five post-labelling delays (PLD; 0.2, 0.7, 1.2, 1.7 and 2.2 s); 43 label and 43 control images; 2 fully relaxed M0 images; Acquisition time (TA) 5:29; Repetition time (TR) 3580 ms; Echo time (TE) 19 ms; Flip Angle (FA) 90; Field of view (FOV) 256 × 256 mm; Matrix 86 × 86; Multi-slice acceleration factor 6. The Siemens 3 T Prisma scanner at the Royal Children’s Hospital underwent a necessary software update from the VE11C operating system to the XA30 operating system in December 2022, which necessitated a change in the ASL sequence. The scans acquired following the update (n = 45, VP: 24, Controls = 21) are not included in the current analysis due to the complexity of integrating the markedly different acquisition parameters.

### Image analysis

ASL images were analyzed using an established pipeline previously applied in a paediatric cohort^20^. All ASL PLDs were utilised, except for the 0.2 and 0.7 s PLD due to a mismatch with arterial transit time (i.e., the blood would have not reached the tissue in time to accurately measure perfusion). The ASL images were motion corrected using an affine transformation^40^, and motion parameters were computed for later analysis (specifically, the root mean square (RMS) translational motion in mm)^20^. Using in-house routines in Interactive Data Language (IDL)^20^, CBF maps (ml/100 g/min) were created using the 2-compartment model^41,42^, as well as literature values^43^ for labelling efficiency, gray matter tissue T1, arterial T1, brain-blood partition coefficient, and tissue transit time. Tissue segmentations of grey matter, white matter and cerebrospinal fluid tissue were obtained based on the ASL control images using Statistical Parametric Mapping version 8 (SPM8) software (see Supplementary Figs. 1, 2 and 3, for samples of the tissue segmentation). Using the gray matter template in SPM8, gray matter segmentations were spatially normalized into Montreal Neurological Institute (MNI) space. A study-specific grey matter template was also created by averaging across participants and spatial normalization repeated^20^. Regional CBF maps were transformed into template space using the same transformation, and spatially filtered using a Gaussian filter (σ = 4 mm, corresponding to FWHM = 9.42 mm)^20^.

### Statistical analyses

As our study aimed to describe relationships between CBF and cognitive outcomes, we did not adjust for potential demographic or preterm-related characteristics that may influence these associations^44^. Participant characteristics were summarised using STATA 17 (StataCorp, 2021), including counts and percentages or means and standard deviations.

Using IDL, all statistical analyses of the CBF maps were performed using voxel-wise general linear models, with every model including age, sex, the RMS motion parameter described above, and grey matter probability. Based on prior work, these analyses were all also restricted to participants with RMS motion < 1 mm, and to voxels with grey matter probability > 78%^20^, which restricted the analyses to grey matter (including cortical and subcortical grey matter). No participants were excluded due to extreme motion. The general linear models were used to compare CBF between the VP and term-born control groups. These models were also used to analyze the relationships between CBF and cognitive outcomes (separate models for each cognitive outcome) following three steps. Firstly, the relationships between CBF and cognitive outcomes were analyzed for the entire cohort (VP and term groups combined). Secondly, birth group-by-cognitive outcome interaction terms were included to determine whether CBF and cognitive outcome relationships differed between birth groups. Finally, the relationships between CBF and cognitive outcomes were analyzed separately for the VP and term-born groups, to explore these relationships in each birth group separately. Model equations are provided in the Supplementary Material. Using in-house software in IDL, all analyses were corrected for voxel-wise multiple comparisons using a Monte Carlo analysis^45^ with statistical significance defined as Family-Wise-Error (FWE)-corrected* p* value < 0.05. Specifically, autocorrelation was estimated from the residual maps, and noise images were generated based on this autocorrelation, with exogenous spatial filtering also applied. A total of 1000 iterations were performed. Anatomical localisation of significant voxels was aided by visual inspection and the Automated Anatomical Labelling (AAL) atlas^46^. Following a similar approach to previous ASL studies^20,47^, voxel-wise results are reported as *T*-values, representing the degree of difference in significant voxels.

## Results

### Sample characteristics

Of the 136 VP and 46 controls who participated in the 20-year follow-up, MRI scans were performed on 107 VP and 40 term-born controls. Of these, 79 VP and 16 term-born controls were scanned with the original HCP ASL sequence prior to the scanner upgrade. Based on subjective visual assessment, ASL images for 6 VP individuals were of poor quality and were removed prior to analyses (none of these were excluded due to motion, as motion-corrupted frames were removed), leaving 73 VP adults with high-quality ASL data. The final sample comprised 72 participants born VP and 16 participants born at term with high-quality ASL data who also participated in the cognitive assessment (refer to supplementary Fig. 4 for a detailed flowchart).

There were minimal differences in medical or sociodemographic characteristics between study participants and non-participants, with the exception of higher social risk in non-participants compared with participants in both birth groups (Supplementary Table 2).

Neonatal and sociodemographic characteristics of the study birth groups are presented in Table 1. Age and sex were similar between the VP and FT groups, but there were more participants with higher social risk in the VP group compared with term-born controls.

**Table 1:** Perinatal and sociodemographic characteristics of participants with ASL and cognitive data at 20 years of age.

Variable	VP *N* = 72	FT *N* = 16
Sex (M), n (%)	36 (50)	8 (50)
Age at cognitive assessment (years), M (SD)	20.1 (0.5)	19.8 (0.4)
Perinatal medical variables		
Multiple birth (twin or triplet), n (%)	36 (50)	0 (0)
Gestational age (weeks), M (SD)	27.4 (2.1)	39.1 (1.3)
Birthweight (g), M (SD)	963 (220)	3331 (420)
Grade 3 or 4 IVH, n (%)	2 (2.8)	0 (0)^a^
Moderate to severe white matter injury, n (%)	15 (20.8)	0 (0)^a^
Cystic PVL, n (%)	4 (5.6)	0 (0)^a^
Bronchopulmonary dysplasia, n (%)	21 (29.2)	0 (0)^a^
Surgery in the newborn period, n (%)	24/68 (35.3)^b^	0 (0)
Sociodemographic variables		
Higher social risk* at 13 years, n (%)	32/67 (47.8)^c^	2/15 (13.3)^d^
^¶^20-year cognitive outcomes *M (SD)*		
IQ	0.24 (1.0)	0.48 (0.7)
Receptive language	− 0.06 (0.9)	0.37 (0.6)
Processing speed	0.14 (1.0)	− 0.14 (0.7)
*Verbal learning and memory*		
Verbal immediate memory	0.09 (0.9)	0.18 (0.8)
Verbal learning	− 0.002 (1.0)	0.35 (0.6)
Verbal delayed memory	− 0.07 (1.0)	0.40 (0.7)
*Executive function*		
Working memory	0.16 (1)	0.19 (0.9)
Planning	− 0.02 (0.9)	0.57 (0.9)
Cognitive flexibility	− 0.17 (0.5)	0.44 (0.5)

M = mean; SD = standard deviation; n = number; % = percentage; VP = Very Preterm; FT = Full-term; IVH = intraventricular hemorrhage; PVL = periventricular leukomalacia. *As described in previous follow-ups of this cohort^32,33^, social risk was dichotomised into higher and lower social risk groups based on six sociodemographic factors. ¶ Z-scores are presented for each cognitive outcome Please note that the number of individuals who completed each cognitive test varies. ^a^ = 3 missing; ^b^ = 4 missing; ^c^ = 5 missing; ^d^ = 1 missing.

### Cerebral blood flow differences by birth group

**Fig. 1:**
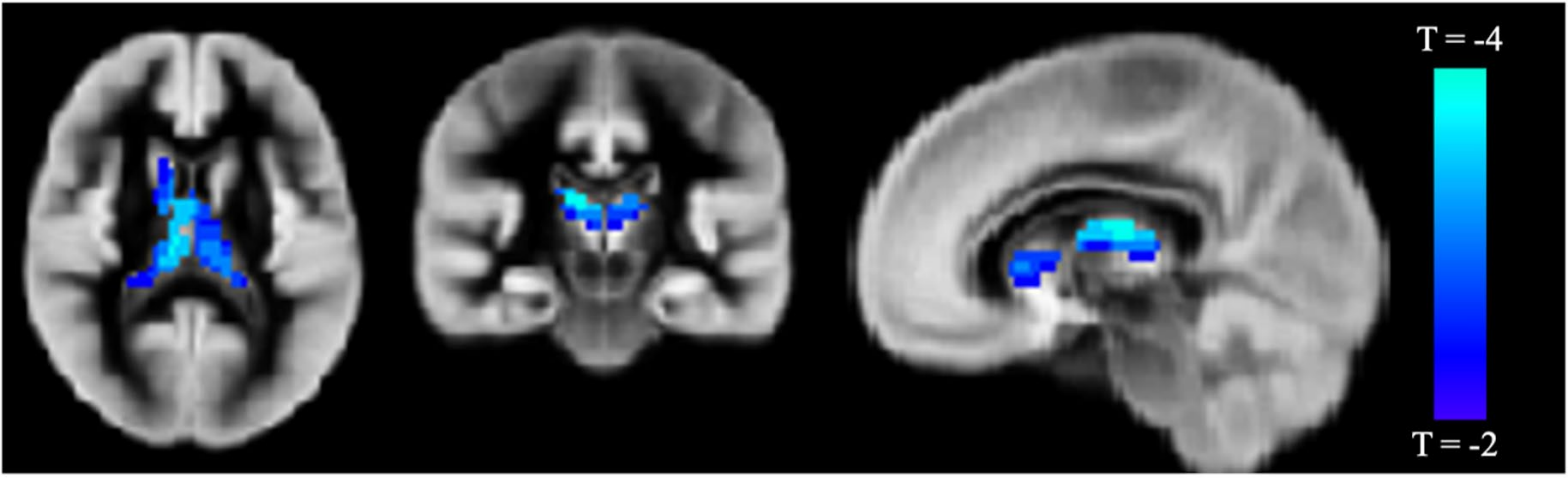
Cerebral blood flow (CBF) compared between VP and term-born groups. Voxels with reductions (FWE-corrected *p* < 0.05) in CBF in those born VP are color-coded and overlaid on the grey matter tissue probability template. T-values represent the magnitude of difference. The images are presented in radiological orientation.

Compared with term controls, young adults born VP displayed reduced CBF in the deep grey matter (including the right caudate and thalamus bilaterally) (Fig. 1, Supplementary Table 3). No other regions (cortical or additional subcortical regions) reached significance after correction for multiple comparisons.

### Relationships between cerebral blood flow and cognitive outcomes: both birth groups combined

**Fig. 2:**
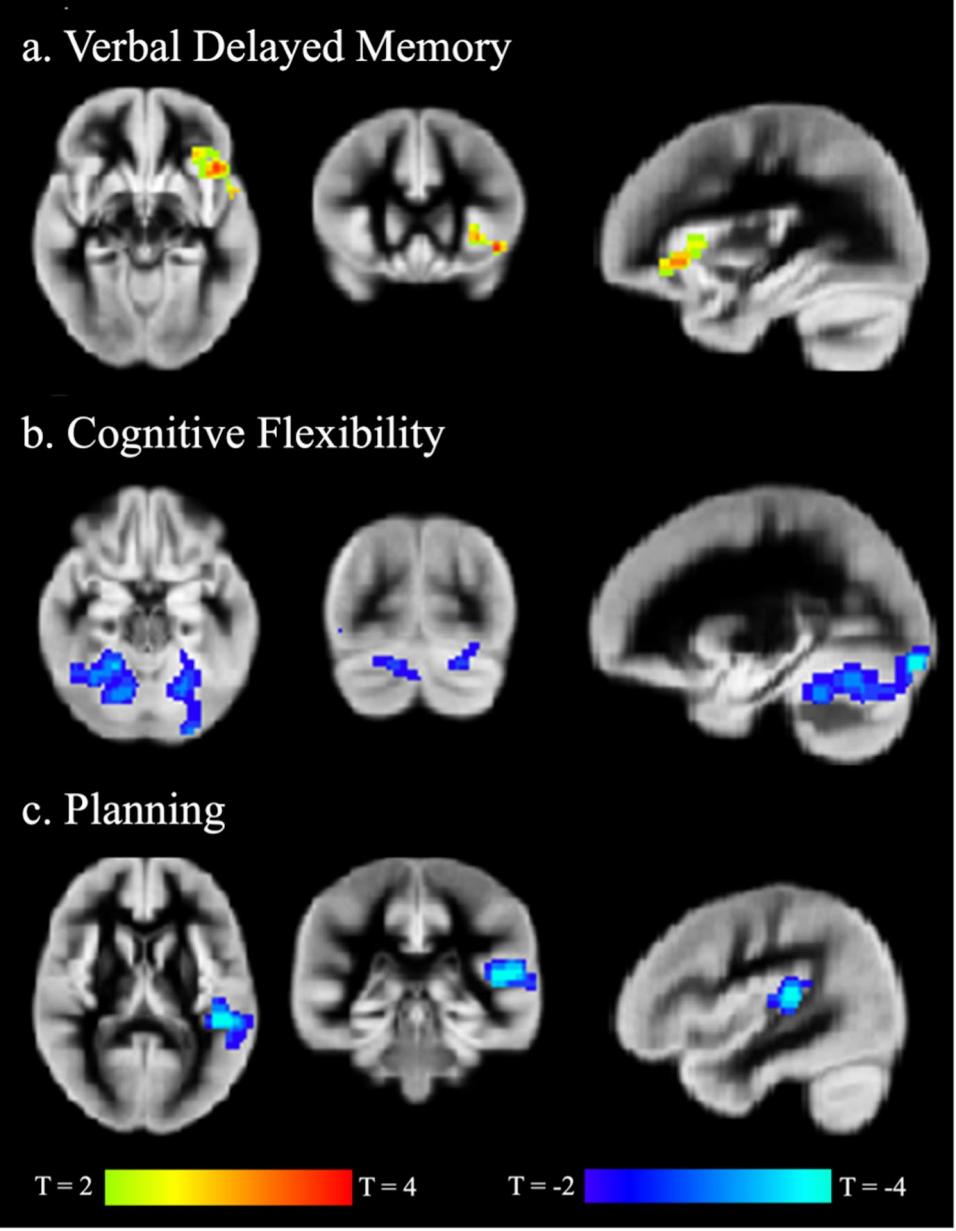
Voxel-wise relationships of cerebral blood flow (CBF) with cognitive outcomes (both groups combined), for (**a**) Verbal delayed memory; (**b**) Cognitive Flexibility; (**c**) Planning. The yellow–red colour bar represents significant positive relationships (FWE-corrected *p* < 0.05) between cerebral blood flow and cognitive outcomes (i.e., better cerebral blood flow relating to better cognitive performance). The dark blue-light blue colour bar represents significant negative relationships (FWE-corrected *p* < 0.05) between cerebral blood flow and cognitive outcomes (i.e., lower cerebral blood flow relating to better cognitive performance). The significant voxels are overlaid on the grey matter tissue probability template. *T*-values represent the direction and strength of significant associations between brain regions and the cognitive outcome. The images are presented in radiological orientation.

Given the large number of brain regions that were significant in the CBF and cognitive outcome analyses, we describe regions by lobe, with specific locations listed in Supplementary Table 4. For both birth groups combined (i.e., in the entire cohort), CBF in the left insula and specific frontal and temporal regions was positively associated with verbal delayed memory, with the largest clusters located in the left insula and inferior orbitofrontal gyrus (Fig. 2a). In contrast, CBF in occipital, temporal and cerebellum regions was negatively associated with cognitive flexibility (Fig. 2b) (i.e., lower CBF related to better cognitive flexibility). The largest clusters were located in the cerebellum, right fusiform gyrus and right inferior temporal lobe. No cerebral regions were associated with IQ, receptive language, processing speed, verbal learning, verbal immediate memory, or working memory.

### Relationships between cerebral blood flow and cognitive outcome by birth group

**Fig. 3:**
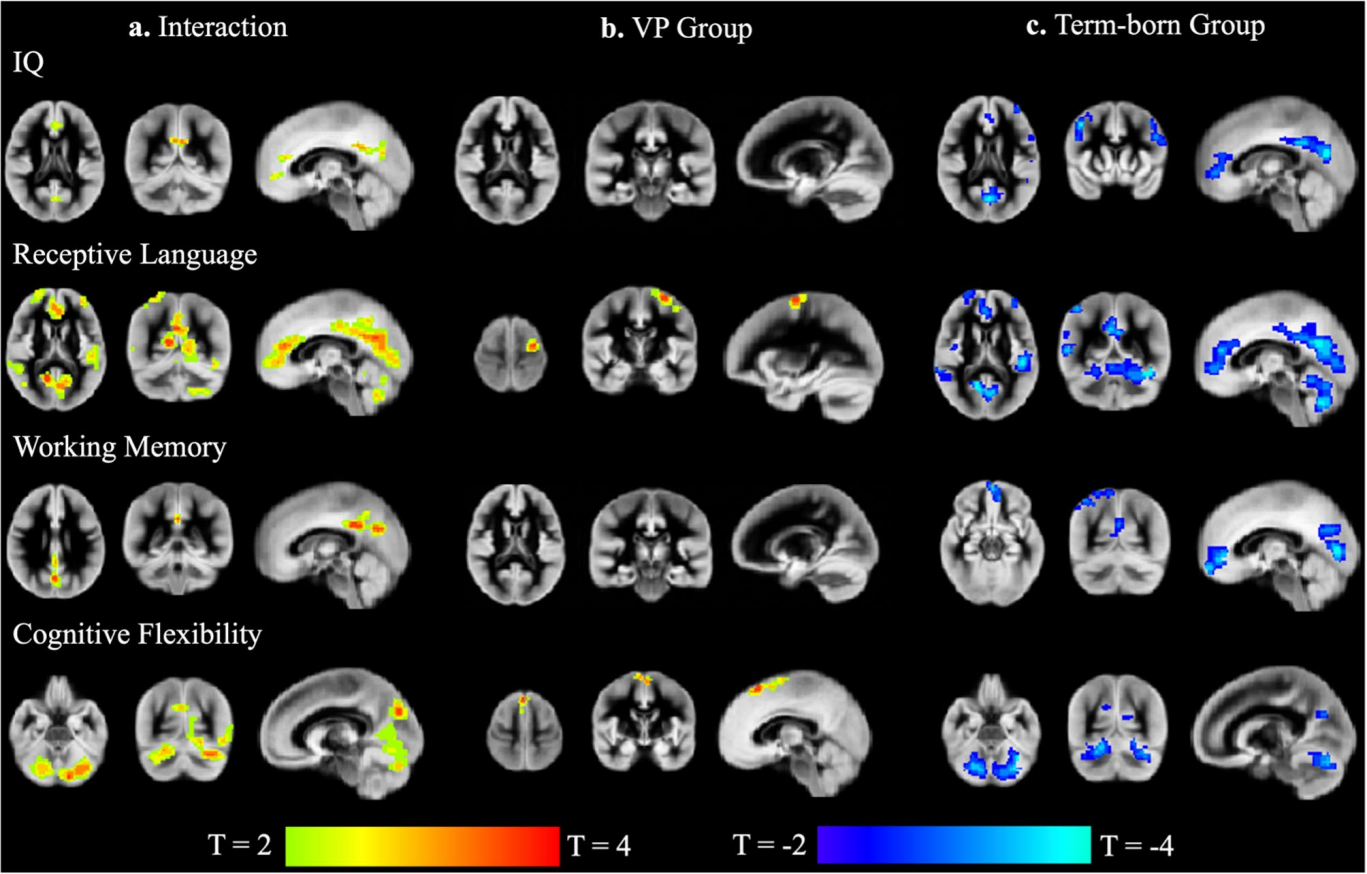
Voxel-wise interactions between cerebral blood flow and birth group on cognitive outcomes, and cerebral blood flow-cognition associations examined separately within the VP and term groups. (**a**) Voxel-wise interactions between cerebral blood flow and birth group for each cognitive outcome for both groups combined. The yellow–red colour bar indicates the presence of a significant interaction (FWE-corrected *p* < 0.05); (**b**) Voxel-wise relationships between cerebral blood flow and each cognitive outcome for the VP group alone; c. Voxel-wise relationships between cerebral blood flow and each cognitive outcome for the term group alone. In parts (**b** and **c**), the yellow–red colour bar represents significant positive relationships (FWE-corrected *p* < 0.05) between cerebral blood flow and cognitive outcome (i.e., higher cerebral blood flow relating to better cognitive performance), while the dark blue-light blue colour bar represents significant negative relationships between cerebral blood flow and cognitive outcomes (i.e., lower cerebral blood flow relating to better cognitive performance). The significant voxels are overlaid on the grey matter tissue probability template. *T*-values represent the direction and strength of significant associations between brain regions and the cognitive outcome. The images are presented in radiological orientation.

The relationships between CBF and cognitive outcomes differed by birth group for IQ, receptive language, working memory, and cognitive flexibility (indicated by the significant CBF-by-group interactions in Fig. 3a). To interpret these interactions, voxel-wise associations are presented separately for the VP and term-born groups in Fig. 3b,c.

IQ was negatively associated with CBF in specific locations across frontal, cingulate, parietal, and occipital regions in the term group (Fig. 3c), with the largest clusters observed bilaterally in the middle frontal gyrus, left calcarine fissure, right superior parietal lobe and left superior temporal lobe. No significant associations between IQ and CBF were identified in the VP group alone (Fig. 3b).

Receptive language was negatively associated with CBF across frontal, insula, cingulate, temporal, parietal, occipital and cerebellum regions in the term group (Fig. 3c). The largest clusters involved in the association included right superior frontal gyrus, bilateral middle frontal gyrus, left anterior cingulum, left calcarine fissure, left lingual gyrus, left fusiform gyrus, right superior parietal gyrus, precuneus bilaterally, right middle and superior temporal gyrus, and parts of the cerebellum. In contrast, receptive language was positively associated with CBF in small number of regions in the VP group (Fig. 3b). Working memory was negatively associated with CBF across cingulate and occipital regions in the term group (Fig. 3c), with clusters in the left medial orbital frontal cortex, left calcarine fissure and right superior parietal lobe. No significant associations were identified in the VP group (Fig. 3b).

Cognitive flexibility was negatively associated with CBF in specific temporal, cingulate, occipital, parietal and cerebellum regions in the term group (Fig. 3c), with the largest clusters involving the cerebellum, left lingual and right fusiform gyrus. The VP group displayed positive relationships in the supplementary motor area and medial part of the superior frontal gyrus bilaterally (Fig. 3b).

While interactions between CBF and verbal immediate memory, verbal learning, and verbal delayed memory did not reach statistical significance when examining the birth groups separately, the term group exhibited negative relationships between CBF in multiple brain regions and verbal immediate memory (Supplementary Fig. 5) and verbal learning (Supplementary Fig. 6), while for the VP group there were positive relationships between CBF in restricted brain regions and verbal delayed memory (Supplementary Fig. 7).

## Discussion

The current study found that CBF in young adults born VP was lower than their term-born peers in selective subcortical regions. In the combined VP and term cohort, lower CBF was related to better cognitive flexibility and planning performance, but reduced verbal delayed memory performance. However, we noted birth group interactions between CBF and IQ, receptive language, working memory and cognitive flexibility, underpinned by negative relationships between CBF and cognition observed in the term group. The few CBF-cognitive relationships observed in the VP group were positive.

Consistent with previous reports of children born VP and young adults born VLBW, we found lower CBF in subcortical regions^47,48^, particularly the thalamus^47^, compared with term-born controls. These findings are also broadly in agreement with reported volumetric, microstructural and morphological alterations across subcortical regions in young adults born VP compared with term-born controls^49^. While our results did not correspond to all subcortical structures reported in previous studies, the collective evidence suggests subcortical regions may be particularly vulnerable to demonstrate lower CBF in individuals born VP. This selective regional vulnerability may reflect a sensitivity “signal” bias in that the thalami represent downstream cortical signalling from much larger cortical regions, which may not reach threshold in this sized cohort for detection. It is well known that both primary injury and secondary degeneration occur from neuronal and white matter injury in the thalami and deep nuclear gray matter in the VP infant^50^. Thus, alterations in CBF in the thalami and deep nuclear gray matter may further reflect this with sufficient sensitivity based on their integrated central nature to reflect global cerebral impact in the very preterm infant.

Birth group differences in CBF were not identified in cortical regions, which may be due to insufficient power to detect more subtle differences and sensitivity of our neuroimaging techniques. Our findings differ from previous reports that have reported higher CBF in specific cortical regions among children and adolescents born VP compared with term-born controls. As CBF increases across early childhood and then decreases thereafter to reach a plateau in early adulthood^51^, the discrepancy may be due to age differences between studies, where participants in Hijman et al. (2024) were aged 12.9 years (mean)^25^ compared with a mean age of 20 years in the current study. Hijman et al. (2024) potentially assessed CBF during a dynamic period of development, while our study assessed CBF during a more stable period of cerebral development.

Our findings add to the growing evidence that CBF is associated with cognitive functioning. In younger populations, there is mixed directionality of relationships between CBF and cognitive outcome^20,48^. While there is evidence of positive associations between CBF and executive function in children and adolescents born VP and at term^48^, and with executive functioning and aspects of IQ in other clinical populations^18,19,52^, there is also support for negative relationships^17,20^, some of which we observed in the current study. Our results are contrary to findings in ageing populations^12,13,53^ where higher CBF is consistently associated with better cognitive performance. Our findings most likely reflect the nature of CBF—cognition relationships observed in young adult populations, which are unaffected by the potential pathophysiological mechanisms of ageing.

Our findings that the relationship between CBF and selected cognitive outcomes differed by birth group are novel, but require substantiation in other cohorts. The stronger negative relationships between CBF and aspects of cognitive functioning in the term group are partially consistent with the limited literature in healthy populations^17,54^. For example, negative relationships between IQ and CBF in temporal regions have been observed in typically developing children^17^, although positive CBF—IQ relationships were also observed in other brain regions. Further research is required to better understand CBF—cognition relationships in healthy populations, providing a reference point for interpreting deviations in clinical populations. Nonetheless, we recognise that a greater number of CBF—cognition relationships were significant in the term-born group compared with the VP group, where we expected to find neurophysiological and cognitive alterations. Based on the limited literature available in the VP population, we can only speculate that greater cognitive and neurophysiological heterogeneity in the VP group, compared with the term-born group, may have reduced the power to detect consistent associations between CBF and cognitive outcomes.

The neural efficiency hypothesis, which proposes that brains of individuals with higher IQ are more efficient and thus require less energy^55–57^, may partially explain the opposing relationships between CBF and cognition in the VP and term groups. However, we acknowledge that this framework typically refers to task-related brain activity and is moderated by task complexity^55,58,59^, such that individuals with higher IQ display *greater* brain activation on more difficult tasks^55^. In our study, negative associations in the term-born group were most consistently found in the medial default mode network, rather than task-related networks (e.g., fronto-parietal networks), likely reflecting that cognitive functioning and CBF measurements were not measured concurrently. Considering task complexity, we may have also expected positive relationships in the term group for the more demanding executive function tasks. Inter-individual differences (e.g., different individuals will find different tasks easy or hard) should also be noted.

Another potential explanation for our findings lies in the development of CBF. CBF increases across childhood and decreases thereafter^51,60^, a trajectory that is also observed in the maturation of cortical thickness and grey matter volume^61^. Thus, the negative relationships observed in the term group may reflect typical brain maturation, where lower CBF reflects a more ‘mature’ brain, and by extension, more efficient cognitive functioning^62,63^. The overall lack of relationships between CBF and cognitive outcome in the VP group may potentially reflect delayed maturation or dysmaturation, as has been suggested in studies examining cortical thickness in individuals born VP^49,62,64,65^. While cerebral dysmaturation is a possible explanation for our findings, it is important to acknowledge that our findings are correlational in nature, and comparisons with the term-born group should be interpreted with caution due to the smaller sample size (*N* = 16). Further research is required to replicate our findings in larger samples.

Our findings potentially have clinical implications. Namely, those born VP do not display the same CBF—cognition relationships as their term peers, contributing to the literature on altered brain architecture in the VP group and elucidating CBF as an additional marker of developmental differences in brain function^49^. While altered haemodynamic relationships did not underpin cognitive difficulties in the VP group, persistent cognitive and behavioral difficulties^6,66–68^, along with altered brain structure and function in adults born VP^49^, merit ongoing monitoring. Furthermore, given the limited literature into long-term cognitive outcomes in late adulthood for this population, it is plausible that differences in resting-state brain function in young adulthood may provide early insight into ageing-related vulnerability. Lastly, we found no evidence of ‘catch-up’ in brain maturation for young adults born VP compared with their term peers^69,70^.

A strength of our study was the assessment of multiple cognitive domains. While previous studies have used composite scores^20,48^ or global measures of cognitive functioning^17^, the current study also explored specific cognitive skills hypothesised to be associated with CBF. Additionally, the use of ASL with multiple post-labelling delays offers a direct and reliable quantification of CBF^9^, strengthening the interpretation of our findings. Lastly, our exploration of brain-behavior relationships adds to the relatively limited literature on the neural underpinnings of cognitive outcomes in young adults born VP^71–74^.

We acknowledge our small and unbalanced sample sizes, limiting comparability across birth groups and reducing precision in estimates between CBF and cognitive outcomes. Further research with larger samples is required to confirm our findings. While exploration of potential demographic confounders and subgroups within the VP group was beyond the scope of the current study and limited by our sample size, future research with larger samples may explore whether these factors influence relationships between CBF and cognitive outcomes. Our relatively low retention rate at the 20-year time-point may have introduced selection bias in our findings, with implications for generalisability. While demographic differences between participants and non-participants at 20-years were minimal, the findings may not generalise beyond the demographic and clinical characteristics of the VIBeS cohort. We also recognise that we used cognitive outcome scores that are not completely independent, an inherent limitation of neuropsychological assessments. Lastly, our study is cross-sectional, and therefore cannot define the temporal sequence nor the causal nature of the relationships between CBF and cognitive outcomes.

Future research incorporating CBF with structural and functional outcomes may provide a more nuanced picture of the neural architecture in young adults born VP, and how it relates to cognitive functioning. There is also growing recognition that brain and cognitive health intersect with cardiovascular functioning^75^. It may be of interest to understand whether CBF—cognitive relationships are modified by different cardiovascular profiles, particularly in populations who are at greater risk for poorer cardiovascular health, such as those born VP^76^.

CBF in selective subcortical regions is lower in those born VP compared with term peers, and the relationship between CBF and cognition differed between these two groups. These findings extend our understanding of brain architecture in young adults born VP, indicating cerebrovascular differences that may be consistent with delayed or atypical neurovascular maturation. Future longitudinal studies are needed to determine whether these differences persist and influence cognitive aging outcomes.

## Supplementary Information

Below is the link to the electronic supplementary material.

Supplementary Material 1

## Data Availability

Data are available for the current study upon reasonable request to the corresponding author, subject to ethical approval and adequate data sharing agreements.
