# Supplementary material for: Relationships between cerebral blood flow and cognition in young adults born very preterm and at term
Source: Sci Rep. 2026 Apr 21;16:18434. doi: 10.1038/s41598-026-49390-6 (PMC13265763; doi:10.1038/s41598-026-49390-6)
Supplement: Supplementary file 1 — Supplementary Material 1 [file 41598_2026_49390_MOESM1_ESM.pdf]

Supplementary Methods

Model equations:

1. Comparison of CBF between very preterm and term groups:

$$CBF = A0 + A1*[group] + A2*[sex] + A3*[age] + A4*[GM] + A5*[motion] + N[0, s^2]$$

2. Relationships between CBF and cognitive outcomes in the entire cohort (very preterm and term):

$$CBF = A0 + A1*[group] + A2*[sex] + A3*[age] + A4*[GM] + A5*[motion] + N[0, s^2] + A6*[cognitive]$$

3. Relationships between CBF and cognitive outcomes in the entire cohort, including a group (very preterm or term) interaction:

$$CBF = A0 + A1*[group] + A2*[sex] + A3*[age] + A4*[GM] + A5*[motion] + N[0, s^2] + A6*[cognitive] + A7*[cognitive*group]$$

4. Relationships between CBF and cognitive outcomes, separately for each group (very preterm and term):

$$CBF = A0 + A1*[sex] + A2*[age] + A3*[GM] + A4*[motion] + N[0, s^2] + A5*[cognitive]$$

Supplementary Tables

Table S1. Summary of Cognitive Outcomes Administered at the 20-year follow up.

Domain	Measure	Outcome Variable
**General Intellect**		
General Cognitive Function (IQ)	Kaufman Brief Intelligence Test, second edition (KBIT-2).[1] Three subtests measuring verbal (e.g., receptive vocabulary, general knowledge) and non-verbal (visual reasoning and problem solving) intelligence.	IQ Standard Score<br><br>Receptive Language (using the Verbal Knowledge subtest).
**Processing Speed**	Cogstate: Identification Test (CogState Ltd, Melbourne, Australia)	The mean of the log10 transformed reaction times for correct responses (lower score represents better performance).
**Verbal Episodic Memory**		
Verbal Learning and Memory	The California Verbal Learning Test, third edition (CVLT-3).[2]	*Verbal Immediate Memory*: number of correctly recalled items for trial 1<br>*Verbal Learning:* total of correctly recalled items from trials 1-5.<br>*Verbal Delayed Memory*: number of correctly recalled items after a 20 – 30-minute delay (i.e., long delay free recall).
**Executive Function**		
Cognitive Flexibility (the ability to flexibly shift between task demands)	Contingency Naming Test[3]	Efficiency scores from trial 4.
Working Memory (to hold and manipulate information in mind)	Digit Span Backwards[4]	The sum of correctly recalled trials.

Planning<br>(process of completing steps to reach a goal)	D-KEFS Tower Test[5]	Total Achievement Score (range 0 to 30. Based on the number of moves completed for each correctly built tower within a specific time-limit for each item.

Table S2. Summary characteristics for young adults who had ASL and cognitive data at 20 years and those who did not.

	VP ASL Data		Term-born ASL Data	
	Participant *N*= 72	Non-participant *N*= 152	Participant *N*= 16	Non-participant *N*= 60
Male sex, *n (%)*	36 (50)	78 (51.3)	8 (50)	29 (48.3)
Gestational Age (weeks), *M(SD)*	27.4 (2.1)	27.5 (1.8)	39.1 (1.3)	39.2 (1.3)
Birthweight (g), *M(SD)*	963 (220)	960 (228)	3331 (420)	3316 (542)
Multiple birth, *n (%)*	36 (50)	58 (38.16)	0 (0)	4 (6.7)
Bronchopulmonary dysplasia, *n (%)*	21 (29.2)	54 (35.8)	0 (0)	0 (0)
Surgery in the newborn period, *n (%)*	24 (35.3)[a]	41(29.1)[b]	0 (0)	0 (0)
IVH Grade 3 or 4, *n (%)*	2 (2.78)[a]	6 (4.0)	0 (0)	0 (0)
White matter injury (moderate or severe), *n (%)*	15 (20.8)	29 (19.3)[c]	0 (0)	0 (0)
Cystic PVL, *n (%)*	4 (5.6)	5 (3.3)	0 (0)	0 (0)
Higher Social Risk at 13 years, *n (%)*	32 (47.8)[d]	68 (66.0)[e]	2 (13.3)[f]	19 (42.2)[g]

Note. IVH: Intraventricular haemorrhage grade 3 or 4; PVL: periventricular leukomalacia. [a]*N*= 68; [b]*N*= 141; [c] *N*= 150; [d]*N*= 67; [e]103; [f]*N*= 15; [g] *N*= 45

Table S3. Group differences in cerebral blood flow (ml/100 g/min) for brain regions that were significant in the voxel-wise analysis.

Brain region	VP *M(SD)*	Control *M(SD)*	Mean difference	95% CI
Left Thalamus	54.60 (15.56)	63.09 (15.69)	-9.09	-17.35, -.83
Right Thalamus	53.95 (17.01)	63.19 (14.58)	-10.17	-18.92, -1.43
Right Caudate	52.71 (15.94)	62.66 (17.40)	-10.32	-19.18, -1.45

Note. M= mean; SD= standard deviation. Brain regions were defined using the AAL atlas.[6] Values represent the mean CBF averaged over all voxels in a cluster. To derive these values, a brain mask was applied to the significant clusters and included all voxels within the cluster that overlapped with the corresponding anatomical region defined by the mask.

Table S4. Significant brain regions for cerebral blood flow and cognitive outcome relationships

Cognitive Outcome	Brain Region
	**Both birth groups combined**
Verbal delayed memory	*Left Hemisphere:* insula, inferior orbitofrontal gyrus and superior temporal pole
Cognitive Flexibility	*Bilateral:* cerebellum, lingual gyrus, inferior occipital gyrus, fusiform gyrus<br>*Right Hemisphere:* inferior temporal gyrus
Planning	*Left Hemisphere:* superior and medial temporal gyri, Rolandic operculum
	**Birth Group x Cognitive Outcome Interaction**
IQ	*Bilateral:* anterior cingulate, precuneus<br>*Left Hemisphere:* Orbital region of the medial frontal gyrus, posterior cingulate, calcarine, cuneus
Receptive Language	*Bilateral:* superior and middle frontal gyrus, orbital region of inferior frontal gyrus, triangular region of inferior frontal gyrus, medial frontal gyrus, median and anterior cingulate and paracingulate gyri, left posterior cingulum; inferior, middle and superior temporal gyri, precuneus, post central gyrus, inferior occipital gyrus, lingual gyrus, fusiform gyrus, cuneus, calcarine fissure, cerebellum<br>*Right hemisphere:* insula, precentral gyrus, orbital region of the superior frontal gyrus, opercular region of the inferior frontal gyrus, temporal pole of the superior and middle temporal gyri, inferior and superior parietal gyri, angular gyrus, middle and superior occipital gyri<br>*Left hemisphere:* orbital region of the middle frontal gyrus, Rolandic operculum, orbital region of medial frontal gyrus, right insula, posterior cingulum, supramarginal gyrus, Heschl's gyrus
Working Memory	*Bilateral:* median cingulate and paracingulate gyri, precuneus<br>*Left hemisphere:* posterior cingulate, cuneus
Cognitive Flexibility	*Bilateral:* cuneus, precuneus, cerebellum

	Left hemisphere: middle and inferior temporal gyri, posterior cingulum, calcarine, fusiform, lingual, superior occipital and inferior occipital gyri

Supplementary Figures

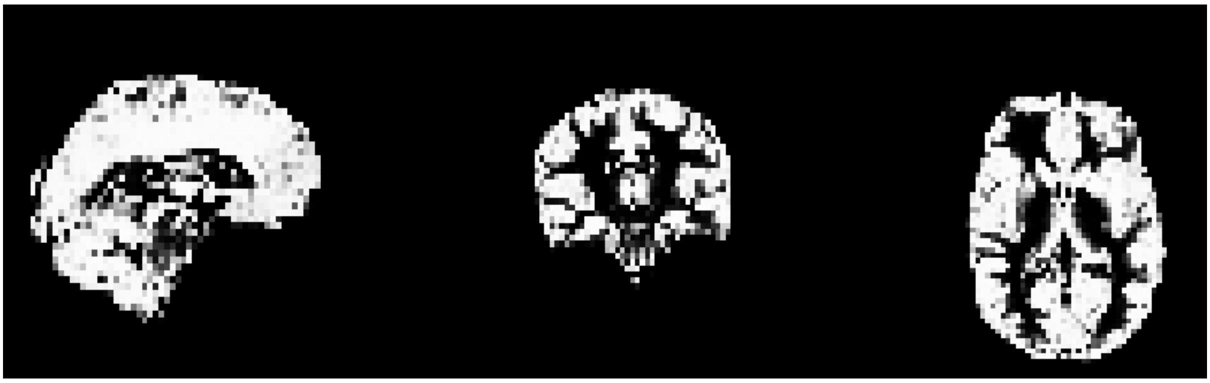

Figure S1. Tissue segmentation of the grey matter from a participant chosen at random.

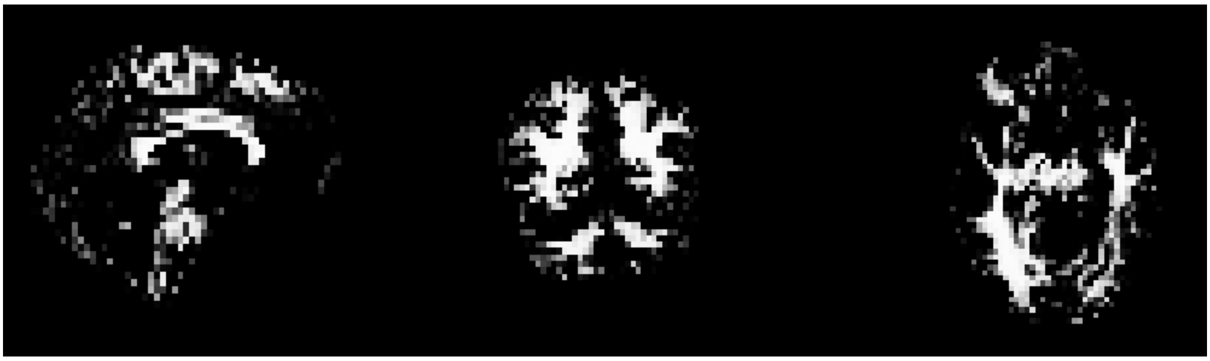

Figure S2. Tissue segmentation of the white matter from a participant chosen at random.

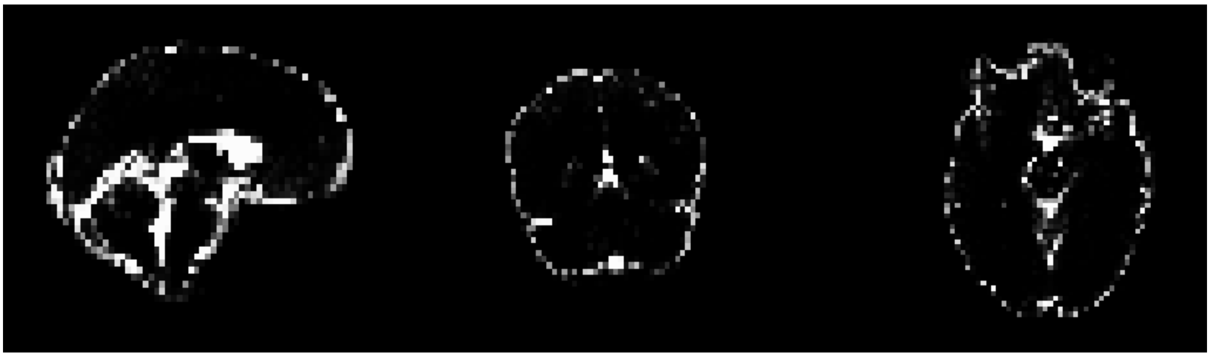

Figure S3. Tissue segmentation of the cerebrospinal fluid from a participant chosen at random.

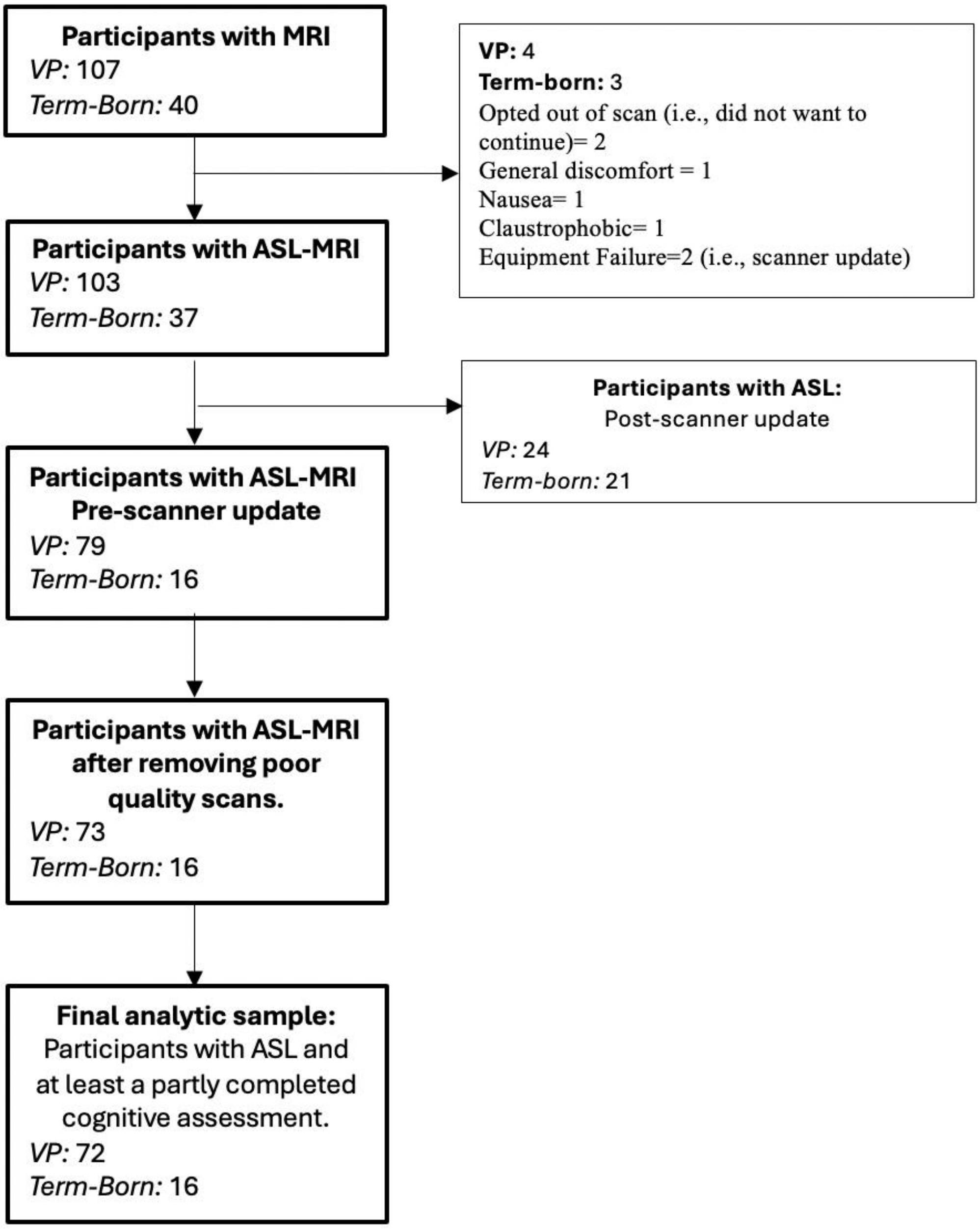

Figure S4. Flowchart presenting sample selection process for the final analytic sample.

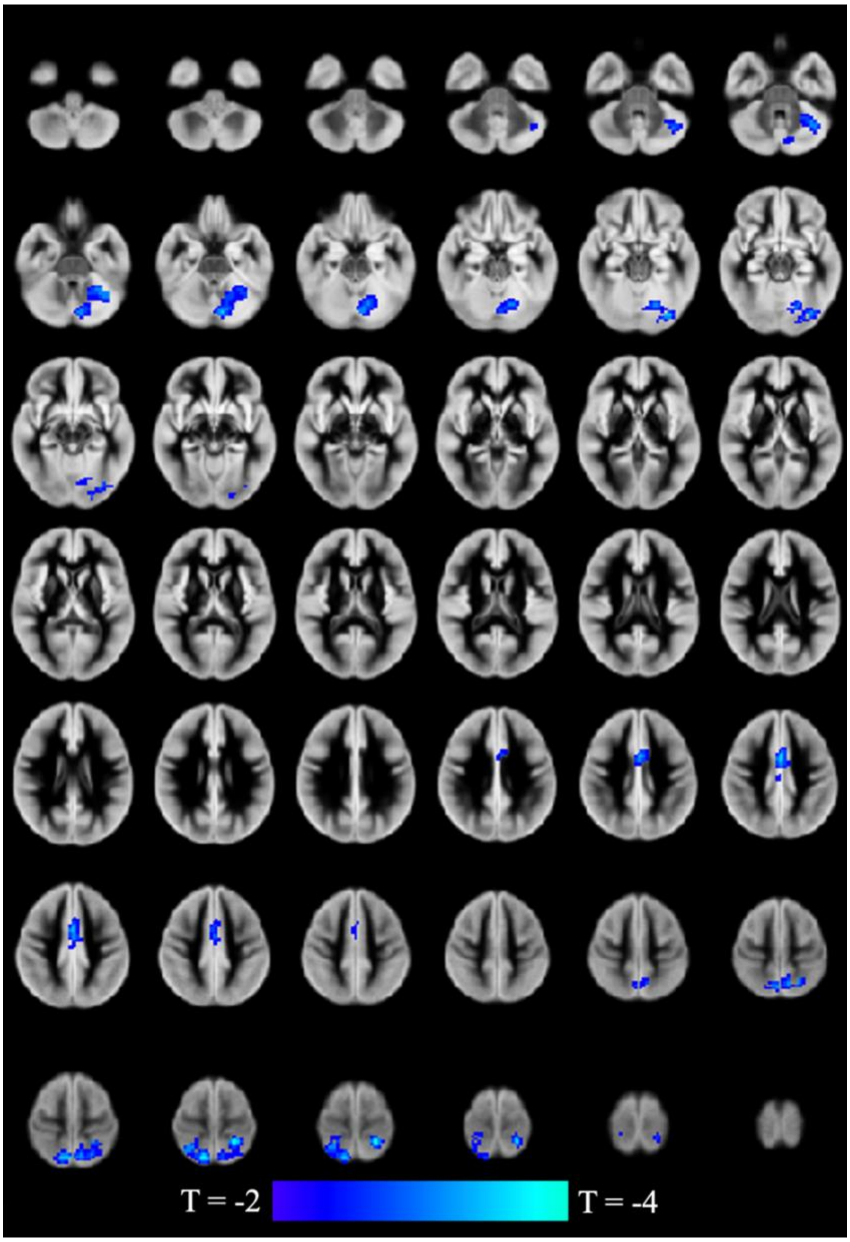

Figure S5. Voxel-wise relationships of cerebral blood flow (CBF) with verbal immediate memory in the term-born group. The dark blue-light blue colour bar represents significant negative relationships (FWE-corrected $p < 0.05$) between cerebral blood flow and cognitive outcomes (i.e., lower cerebral blood flow relating to better cognitive performance). The significant voxels are overlaid on the grey matter tissue probability template. *T*-values represent the direction and strength of significant associations between brain regions and the cognitive outcome. The images are presented in radiological orientation. Significant brain regions: Bilateral: median cingulate and paracingulate gyri, postcentral gyrus, superior parietal gyrus, and precuneus; Left hemisphere: supplementary motor area, lingual gyrus, inferior occipital gyrus, fusiform gyrus, and regions of the cerebellum.

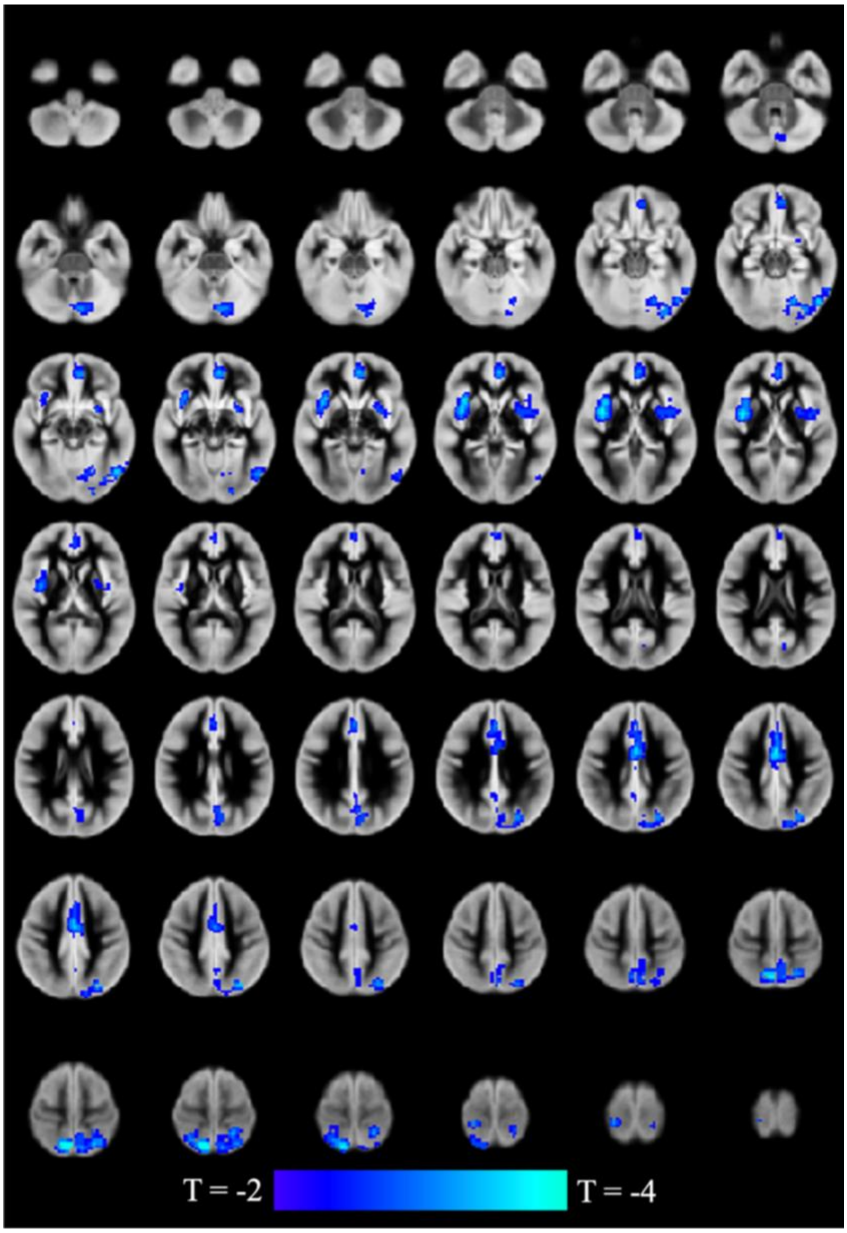

Figure S6. Voxel-wise relationships of cerebral blood flow (CBF) with verbal learning in the term-born group. The dark blue-light blue colour bar represents significant negative relationships (FWE-corrected $p < 0.05$) between cerebral blood flow and cognitive outcomes (i.e., lower cerebral blood flow relating to better cognitive performance). The significant voxels are overlaid on the grey matter tissue probability template. *T*-values represent the direction and strength of significant associations between brain regions and the cognitive outcome. The images are presented in radiological orientation. Significant brain regions: Bilateral: supplementary motor area, insula, median cingulate and paracingulate gyri, postcentral gyri, superior parietal gyri, precuneus and putamen; Left hemisphere: Rolandic operculum, superior frontal gyrus (medial), orbital part of medial frontal gyrus, gyrus rectus, anterior cingulate and paracingulate gyrus, cuneus, lingual gyrus, occipital gyri (inferior, middle and superior), fusiform gyrus, inferior parietal gyrus, inferior temporal gyrus and regions of the cerebellum.

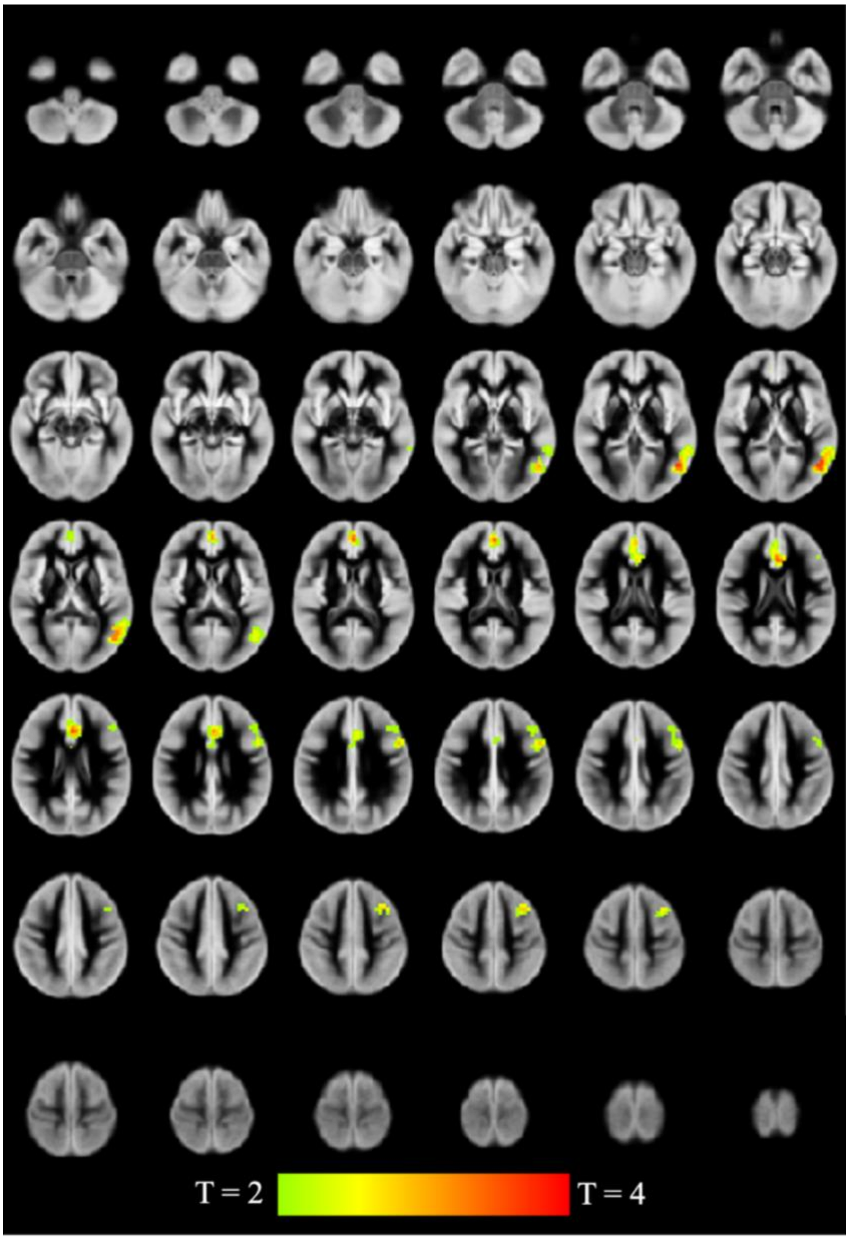

Figure S7. Voxel-wise relationships of cerebral blood flow (CBF) with verbal delayed memory in the VP group. The yellow-red colour bar represents significant positive relationships (FWE-corrected $p < 0.05$) between cerebral blood flow and cognitive outcomes (i.e., better cerebral blood flow relating to better cognitive performance). The significant voxels are overlaid on the grey matter tissue probability template. *T*-values represent the direction and strength of significant associations between brain regions and the cognitive outcome. The images are presented in radiological orientation. Significant brain regions: Bilateral: anterior cingulate and paracingulate gyri; Left hemisphere: precentral gyrus, middle frontal gyrus, opercular and triangular regions of the inferior frontal gyrus, superior frontal gyrus (medial), median cingulate and paracingulate gyri, middle occipital gyrus and middle temporal gyrus.

References

1 Kaufman A, N. K. Kaufman Brief Intelligence Test Second Edition (K-BIT 2). Bloomington, MN: Pearson Assessments. (2004).

2 Delis D, K. J., Kaplan E, Ober B. . California Verbal Learning Test, 3rd Edition (CVLT3). *San Antonio, TX: The Psychological Corporation.* (2017).

3 Anderson, P. J., Anderson, V., Northam, E. A. & Taylor, H. G. Standardization of the contingency naming test (CNT) for school-aged children: a measure of reactive flexibility. **1**, 247-273 (2000).

4 Gonzalez, L. M. Memory function in children with lesions temporal lobe epilepsy and their peers. PhD dissertation. Uof M. (2005).

5 Delis, D. C., Kaplan, E., & Kramer, J. Delis Kaplan Executive Function System. San Antonio, TX: The Psychological Corporation. (2001).

6 Tzourio-Mazoyer, N. *et al.* Automated anatomical labeling of activations in SPM using a macroscopic anatomical parcellation of the MNI MRI single-subject brain. *Neuroimage* **15**, 273-289 (2002). https://doi.org/10.1006/nimg.2001.0978
